# Cranial Morphology of Lithuanian Indigenous Wattle Pigs and Their Hybrids with Wild Boar

**DOI:** 10.3390/ani13091453

**Published:** 2023-04-24

**Authors:** Violeta Razmaitė, Artūras Šiukščius, Šarūnė Marašinskienė

**Affiliations:** Department of Animal Breeding and Reproduction, Animal Science Institute, Lithuanian University of Health Sciences, R. Žebenkos 12, 82317 Baisogala, Lithuania

**Keywords:** *Sus scrofa*, domestic, wild, skull

## Abstract

**Simple Summary:**

Domestic pig hybridization with wild boar occurs for different reasons and needs. The ability to distinguish hybrids is important for monitoring of hybrid and purebred populations and for conservation of both rare domestic pig breeds and wild boars. As cranial morphology has a good discrimination power, the aim of this study was to quantify the differences of cranial morphologies between local Lithuanian Indigenous Wattle pigs and their hybrids with wild boar, including first generation hybrid (domestic x wild) animals and second generation animals (backcross of first generation females with domestic and wild males). Most of the craniometric measurements of hybrids were larger than those of purebred local Lithuanian Indigenous Wattle pigs. A particularly pronounced significant elongation of skull length parameters was found in hybrids. Cranial morphology can be used to discriminate between Lithuanian Indigenous Wattle pigs and their hybrid groups for the all measurements of whole crania and for measurements of distinct parts of crania.

**Abstract:**

The diversity of domestic pig breeds and their hybridization increases the variety of phenotypes expressed in hybrids. The aim of this study was to quantify the differences of cranial morphologies between local Lithuanian Indigenous Wattle pigs and theirhybrids with wild boar. A total of sixteen craniometric measurements were performed on the lateral, ventral and dorsal sides of 71 skulls of Lithuanian Indigenous Wattle pigs and their hybrids, including 1/4 wild boar (WB), 1/2 wild boar and 3/4 wild boar genotypes. The weight of the skull was affected by the genotype, live weight and sex of the animal. The size of the skull, particularly related to skull length parameters, increased consistently with the increase of the wild boar proportion in the hybrids. However, the *Sus scrofa* genotype did not affect the skull height. Clear discrimination was possible between the local Lithuanian breed pigs and their hybrids with different proportions of wild boar and between individual groups of hybrids. The most correct classification was determined on the basis of the overall and length parameters of the crania. This could contribute to better management and utilization of hybrids.

## 1. Introduction

The Eurasian wild boar (*Sus scrofa*) is distributed over most of the world except for very cold and very dry regions [1,2] and has demonstrated a large growth during the last decades in Europe, both in terms of population abundance and distribution range. It has become one of the most abundant wild ungulate animals in Europe [3,4,5], even reaching situations of overabundance in some areas [6,7]. Wild boar as an ancestor of domestic pigs (*Sus scrofa domestica*) can successfully crossbreed with domestic pigs. Hybridization between wild boar and domestic pigs can occur intentionally or accidentally [8]. However, wild boar hybridization with domestic pigs seems to occur at a very low frequency under natural conditions, though it is a common practice in farmed stocks. Through the release of captive hybrid animals [9], it could represent a severe risk for wild populations [9,10]. On the other hand, hybridization between the domestic pig and wild boar seems to have been quite pervasive since domestication [11,12]. These findings were supported by the analysis of different Asian and European wild boar and domestic pig genomes which demonstrated the existence of multiple domestication events and gene flow during and after domestication [13,14,15]. The present genetic divergence between the two forms has mostly developed during the last two centuries when intensive farming has reduced the risk of hybridization in nature [9]. However, new research results suggest that genetic introgression from domestic pigs into wild boar due to varying reasons in different countries may be much more common than expected [5,16,17,18,19]. Wild boar populations with higher domestic ancestry in their genome are mostly concentrated in countries further south than Lithuania [5]; therefore, there is an opinion that Lithuanian free-living wild boars do not contain a domestic pig admixture but this has not been genetically confirmed. Based on personal observation at the exhibition of hunting trophies held in Vilnius, among different trophies a taxidermy of a boar’s head showing a spotted coat color atypical for wild boar was displayed.

Game meat, including wild boar meat, is considered a delicacy and of great quality [20,21,22,23,24,25,26]. It is known that game meat consumption is unequal in different countries. In Europe, game meat consumption is low with only 2–4% of the population consuming this type of meat regularly [27]. This could be explained by the high cost of this type of meat and its greater availability to hunters and their families. Therefore, efforts are being made to rear wild boars in captivity and their hybrids with domestic pigs. Pigs are crossed with wild boar with the aim of improving the taste characteristics of pork, as well as increasing growth rates and the overall production of wild boar in captivity [24]. The ability to clearly distinguish hybrids is very important for monitoring of populations and their conservation, and for avoiding potential fraudulent representation to consumers. Although there are genetic tools to distinguish hybrids from pigs [28,29,30], the acquisition of phenotypic traits remains also important, because breeding of such hybrids relies on the availability of accurate and specific phenotype data. The cranial characteristics of wild boar and the pigs from different areas have been studied [1,31,32,33]. Through their distinct morphologies, wild, domestic and feral pig crania have good discrimination power, demonstrating that wild and domestic pigs can be identified on the basis of cranial morphology [34,35,36,37]. While there have been studies which included both wild boar and domestic pig hybrids [38], data on hybrid cranial characteristics is scarce. Despite the fact that nowadays only a few conventional breeds play an important role in pig production, there are also many rare local pig breeds [39,40], the formation and selection of which have resulted in diversified morphologies.

Distinguishable characteristics between Lithuanian Indigenous Wattle pigs and their 1/4, 1/2 and 3/4 wild boar hybrid’s genotype have been examined through the presence of wattles, coat coloration patterns of newborn piglets and other morphological characteristics based on external body and carcass measurements and anatomical body components of locally slaughtered pigs and their hybrids [41,42].

The aim of this study was to quantify the differences of cranial morphologies between local Lithuanian Indigenous Wattle pigs and their different hybrids with wild boar.

## 2. Materials and Methods

**Figure 1:**
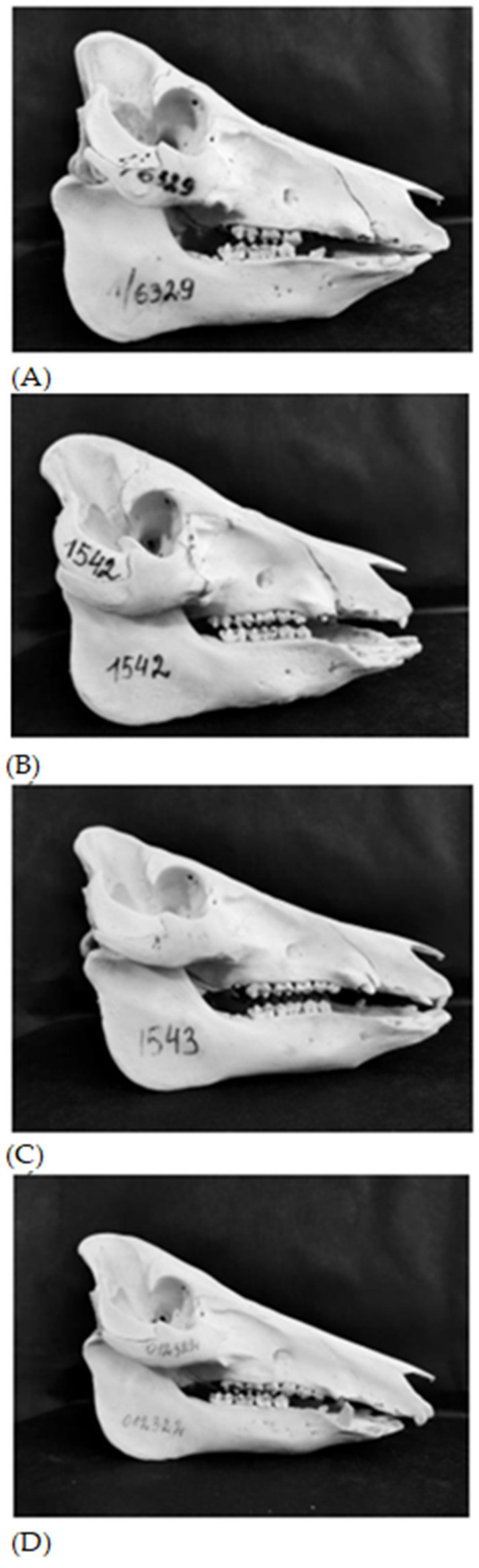
*Sus scrofa* skulls: (**A**) Lithuanian Indigenous Wattle; (**B**) 1/4 WB (1/4 wild boar and 3/4 Lithuanian Indigenous Wattle); (**C**) 1/2 WB (1/2 wild boar and 1/2 Lithuanian Indigenous Wattle); (**D**) 3/4 WB (3/4 wild boar and 1/4 Lithuanian Indigenous Wattle).

A total of 71 skulls were taken from the collection of the Animal Science Institute formed during previous crossing studies. Sixteen skulls belonged to purebred domestic Lithuanian Indigenous Wattle pigs, twenty-one skulls to Lithuanian Indigenous Wattle x wild boar first generation hybrids (1/2 WB genotype), twenty-six skulls to the backcross of 1/2 WB wild boar hybrids with Lithuanian Indigenous Wattle pigs (giving second generation hybrids, with 1/4 WB genotype) and eight skulls to the backcross of 1/2 WB hybrids with wild boar (giving second generation hybrids, with 3/4 WB genotype). Photographs of skulls from all these groups are presented in Figure 1. Purebred Lithuanian Indigenous Wattle pigs were females and castrated males, first generation 1/2 WB genotype and second generation 1/4 WB genotype hybrids were females, entire and castrated males. Second generation 3/4 WB genotype hybrids were females and entire males. The mean weight of live Lithuanian Indigenous Wattle pigs and their hybrids with wild boar of 1/4 WB, 1/2 WB and 3/4 WB genotype before slaughter was 91.5, 88.6, 91.2 and 97.3 kg, respectively.

**Figure 2:**
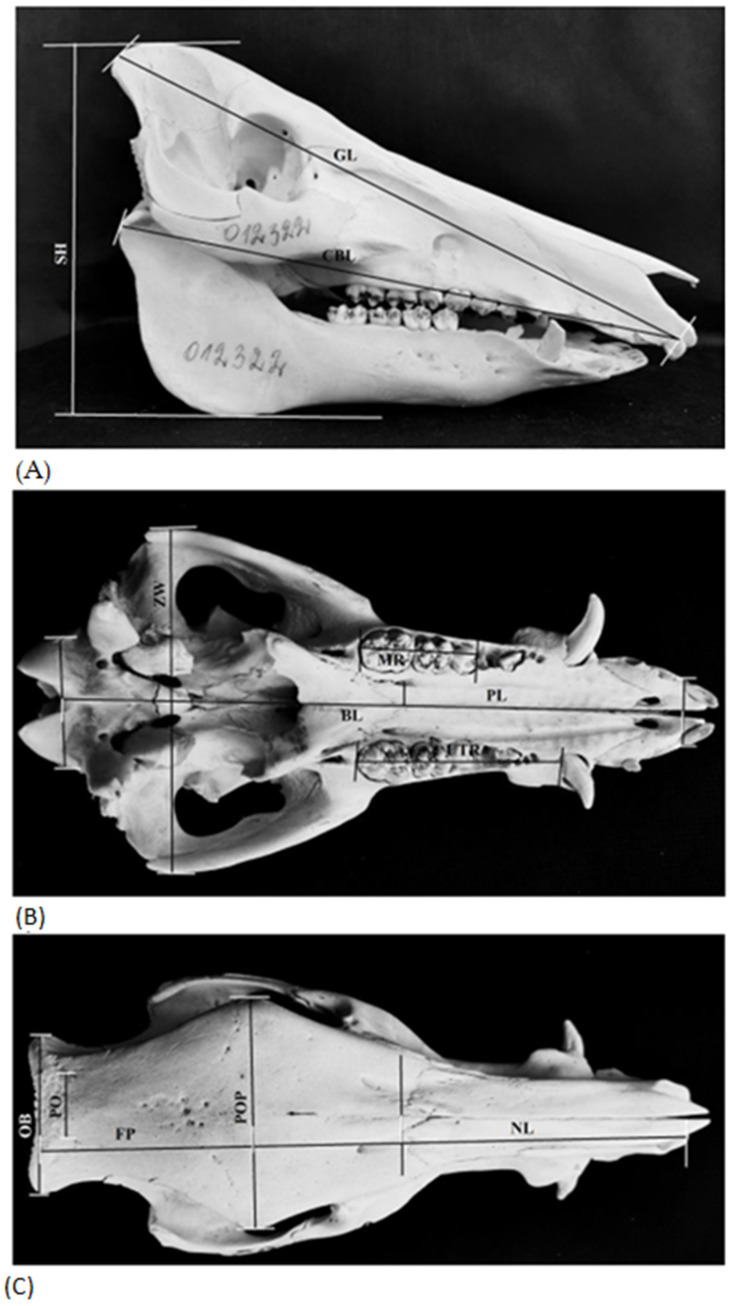
*Sus scrofa* skull diagrams of measurements: (**A**) lateral view-GL-greatest skull length: CBL-condylobasal length; SH-skull height; (**B**) ventral view-BL-basal length: PL-palate length; ZW-bizygomatic width; OB-occipital breadth; UTR-length of upper tooth row; MR-length of upper molar row; (**C**) dorsal view-NL-nasal length: FP-length of frontal + parietal; POP-width across post-orbital processes; PO-post-orbital breadth; OB-occipital breadth.

A total of sixteen craniometric measurements were taken on lateral, ventral and dorsal sides of the skulls (Figure 2). The measurements were taken following Lucchini et al. [43] and Von den Driesch [44].The measurements included: greatest skull length (GL) measured from the opisthocranion, the median point of the line joining the most aboral-dorsal points of cranium; condylobasal length (CBL) measured from the aboral border of the occipital condyles to the prosthion, the median point of the line joining the most oral points of the premaxillae; basal length (BL) measured from the basion, the orobasal border of the foramen magnum in the median plane to the prosthion; bizygomatic width (ZW),the greatest width of skull measured between the most lateral points of the zygomatic arches; occipital breadth (OB), the greatest breadth of the squamous partof the occipital bone; palate length (PL), the distance from the most anterior to the most posterior end of the hard palate; length of upper tooth row (UTR)and length of upper molar row (MR); post-orbital breadth (PO), measured at the least breadth between the temporal lines; width across post-orbital processes (POP), the distance between the most lateral points of the frontal bone on the occipital side of the orbit; frontal + parietal (FP) length; nasal length (NL), the distance from the median point of the naso-frontal suture to Prosthion; occipital height from basion (OHB), measured from the lower ridge of the foramen magnum to the upper side of the occiput; skull height (SH) if placed on its mandible on a flat surface, measured from the highest point on the skull to the surface); length of lower tooth row (LTR), and length of upper incisor row (IR). All measurements were made with an accuracy of 0.1 mm with a pair of Vernier callipers (precision 0.05 mm).

### Statistical Analyses

The data were subjected to multivariate analysis of covariance (MANOVA) in the general linear (GLM) procedure with the least significant difference (LSD) tests in order to determine the differences of estimated marginal (EM) means between the groups. The model included the fixed factors of the *Sus scrofa* genotype and sex, and factor interactions (genotype x sex). The weight of animals was included as a covariate for the determination of animal weight effects on head and skull weights. The weight of skulls was included as a covariate for skull weight effect determination on craniometric features. The differences were regarded as significant when *p* < 0.05. Additionally, discriminant analysis (DA) with leave-one-out cross-validation and principal component analysis (PCA) based on correlation matrices were performed. MANCOVA and DA were performed in IBM SPSS Statistics 27 (IBM, Armonk, NY, USA). PCA was performed in Minitab 15 (Mintab Inc., State College, PA, USA).

## 3. Results and Discussion

### 3.1. Head and Skull Size

The size of the head and skull of the domestic Lithuanian Indigenous Wattle pigs is clearly distinct from the hybrids with wild boar (Table 1). The size of the head and skull increases consistently with the increase of the proportion of wild boar in the hybrids (*p* < 0.001).

Other authors [34,37,45] have indicated that environment has also impact on *Susscrofa* morphology; therefore, comparative morphology can be informative in distinguishing the wild and domestic animals if the issues associated with their size are controlled. In the present study, all the used skulls were from domestic and hybrid animals of similar live weight raised in the same environmental conditions. In addition to the genotype of the animals, their weight and sex also appeared to show an effect (*p* < 0.001) on head weight as well as head weight affecting skull weight. Supplementary data regarding the weight of animals and skull measurements can be found in Table S1. Sex also demonstrated an effect (*p* < 0.01) on skull weight. Sexual dimorphism in the skull size was also observed in other studies [32]; however, the absence of a significant effect of sex and a limited effect of age was reported for feral pigs [37]. In the present study, there was a significant interaction (*p* < 0.05) for skull weight between the genotype and sexof animals. The skull weight of ¼ WB and ¾ WB entire male hybrids was higher than the skull weight of females and castrated males, whereas the skull weight of ½ WB females was higher compared with entire and castrated males. Castrated males of purebred domestic pigs in contrast to hybrids showed lower skull weight than females (Table 1).

### 3.2. Morphological Differences between Groups

The analysis of skull measurements displayed a consistent increase (*p* < 0.001) in the greatest skull length, including condybasal, basal, palate, nasal and frontal + parietal lengths in hybrids with increasing proportions of wild boar genotype (Table 2). Although this study included hybrids with different proportions of wild boar, the findings regarding the measurements of their cranial lengths were in agreement with descriptions of wild boar skulls [34,36,46,47]; this could be explained by the fact that the characteristics inherited from wild males are dominant over the inherited characteristics from domestic females [34].Differences in the *Sus scrofa* genotype did not affect the skull height but the genotype demonstrated an effect (*p* < 0.001) on the occipital height from the basion.

Despite the similarity of these cranial characteristics of hybrids to those of wild boar, the genotype did not show an effect on skull width measurements such as bizygomatic width, width across post-orbital processes, occipital breadth and post-orbital breadth, though wild boars demonstrate not only longer but also slenderer crania compared with domestic pigs [34,36,46].The findings during our previous study [41] on the coat color inheritance of hybrid piglets were consistent with the opinion that the inherited characteristics of the hybrids from wild males are dominant over the inherited characteristics from domestic females [34]; however, this study showed that not all the characteristics were equally inherited, since the hybrids may be forms intermediate between both parents, forms more similar to one parent than the other, or distinct from both parents [38]. Domestic and wild boar crossing has increased (*p* < 0.001) the length of the lower tooth row in all the hybrid groups and also increased (*p* < 0.001, *p* < 0.001 and *p* < 0.01, respectively) the length of the upper tooth row, upper molar and upper incisor rows with the exception of 1/4 WB hybrids. Distinct dental phenotypes also were observed by other authors between wild boars and domestic pigs, as well as among different domestic breeds [31,36,38]. Owen et al. [34] have found that sexual dimorphism was not significant in wild boars but significant in domestic pigs. In our pooled dataset containing more hybrid than purebred domestic pigs, only post-orbital breadth and length of upper molar and lower tooth rows were not affected by sex and skull weight. The skull weight did not show any influence on the skull height and length of upper incisor row. Significant (*p* < 0.05, *p* < 0.001 and *p* < 0.01, respectively) interactions were determined for occipital height from the basion and the length of upper and lower tooth rows between the genotype and sex. Although castrated males in all the groups demonstrated the lowest occipital height from the basion, 1/4 WB hybrid females showed higher, whereas 1/2 WB and 3/4 WB females showed lower, occipital height than entire males. The length of upper and lower tooth rows of 1/4 WB and 3/4 WB hybrid males was higher than that of females; however, the row length of domestic pig and 1/2 WB hybrid females was higher than that of males (Table 2).

The discrimination between purebred domestic pigs and the hybrid groups with wild boar on the basis of all measurements of the whole crania and on partial skull length measurements showed the highest proportions of correctly assigned individuals with 90 and 90.1%, respectively, followed by the nasal (85.9%), height (70.4%) and tooth row (64.3%) cranial sub-sets (Table 3). These data indicated that the accuracy of discrimination between domestic pigs and their hybrids is lower than that between purebred domestic and purebred wild animals. Discriminant functions with leave-one-out cross-validation adopted by other authors [34], correctly classified 100% of domestic and wild specimens. However, these authors also reported that separate cranial sub-sets containing measurements of cranial regions such as nasal, orbit and tooth row demonstrated lower scores compared with cross validation using all measurements.

In cross-validation, the skulls were also classified as the predicted genotype groups. The classification results showed that not all the skulls of the predicted groups were assigned to the groups of each originating genotype group. By whole measurements, skulls of 1/4 WB and 3/4 WB genotype were mostly assigned (92.3 and 87.5%, respectively) to their predicted groups. Purebred Lithuanian Indigenous Wattle pigs probably should be more similar to each other than hybrids, including also the terms of their cranial morphology; however, only 86.7% of purebred domestic pigs were assigned to their own group (Table 3). Similar results for different domestic pig breeds were found by Qwen et al. [34], who have also reported that purebred domestic pigs of different breeds were 75–87.5% correctly assigned to their groups, versus 90% of wild boars. By skull length and height measurements, 1/4 WB (88.5 and 80.8%, respectively) and 1/2 WB (90.5 and 76.6%, respectively) genotype hybrids were mostly assigned to their own groups. The lowest proportions of skulls assigned to their predicted groups were those assigned by tooth row.

**Figure 3:**
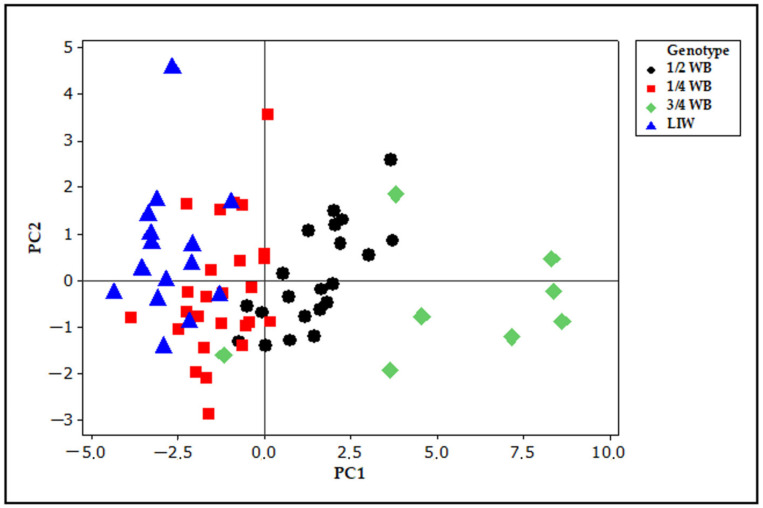
PCA (principal component analysis) score plot of the first principal components (PC1 52.2% and PC2 11.0% of total variance) based on the whole skull measurements for domestic pigs and their hybrids with wild boar.

Principal component analysis (PCA)based on all skull measurements revealed sixteen principal components (PC). However, only for four PC components were the eigenvalues higher than 1.0. The eigenvalues for PC1, PC2, PC3 and PC4 were 8.35, 1.75, 1.43 and 1.08, respectively, and explained of 52.2, 11.0, 8.9 and 6.7% of variance. These first four components accounted for 78.8% of the total variance but the remaining variance was left unexplained. The percentages of variance explained by PCA in Lithuanian Indigenous Wattle pigs and their hybrids with wild boar are lower than those reported by Owen et al. [34] for other different domestic pigs and wild boar. Although PC1 explained the highest percentage of total variance the correlation coefficients between PC1 and all measurements were lower than 0.4. The highest loadings on PC1 have length measurements such as basal length (0.335), palate length (0.334), greatest skull length and condybasal length (0.331). PC2 correlated positively with bizygomatic width (0.547), post-orbital breadth (0.420) and width across post-orbital processes (0.531). PC3 was positively associated with occipital breadth (0.488) and length of upper incisor row (0.453), whereas association with post-orbital breadth was negative (−0.463). PC4 showed positive associations with skull height (0.613) and upper molar row (0.431).Visualization of score plot for the first two PC components is presented in Figure 3.

Although some PC scores of domestic pigs overlapped with those of 1/4 WB hybrids and PC scores of 1/4 WB overlapped with 1/2 WB genotype hybrids, the groups are clearly separated along the PC1 axis with the most distinctive score plot of 3/4 WB hybrids. The measurements of the greatest skull length, including basal, condybasal, palate and nasal lengths, are good descriptors of the skull and mostly contribute to the separation of groups.

## 4. Conclusions

The crania of Lithuanian Indigenous Wattle pigs and their hybrids with wild boar have distinct morphologies. Most craniometric dimensions of different Lithuanian Indigenous Wattle pig hybrid genotypes with wild boar are larger than those of the purebred local Lithuanian pig breed. A consistent elongation of skull length parameters was significant in hybrids having increasing proportions of wild boar, but the changes in width parameters were not significant. Wild boar has increased the length of lower tooth row in all hybrid groups and also increased the length of all upper tooth rows with the exception of 1/4 WB hybrids. The sex of animals and their skull weight did not affect occipital, post-orbital breadth and skull height, or the length of upper molar and of lower tooth rows. The best discrimination values were obtained for whole crania and the length parameters.

Cranial morphology can be used to discriminate hybrids from purebred domestic pigs as well as hybrids with different proportions of wild boar from each other and can contribute to the utilization of hybrids and to the management and conservation of both domestic pigs and wild boar.

## Figures and Tables

**Table 1:** Head and skull weights of different *Sus scrofa* genotypes.

Weight	*Sus scrofa* Groups				*p*-Value			
	LIW	1/4 WB	1/2 WB	3/4 WB	G	S	LW	G x S
Head	4.96 ^c,e^ ± 0.12	5.45 ^d,e^ ± 0.121	5.53 ^d,e^ ± 0.11	7.76 ^f^ ± 0.25	<0.001	0.001	<0.000	0.078
Skull	0.83 ^c,e^ ± 0.02	0.93 ^d,e^′ ± 0.02	1.02 ^f,c^ ± 0.02	1.15 ^f,f^′ ± 0.05	<0.001	0.005	<0.001	0.037

LIW: Lithuanian Indigenous Wattle pigs; 1/4 WB, 1/2 WB and 3/4 WB: LIW hybrids with 1/4, 1/2 and 3/4 proportions of wild boar; G: genotype; LW: live weight; S: sex. *p*-values of GLM LSD tests for genotype mean values within a row with different superscripts differ significantly at ^c,d^ *p* < 0.01; ^e,f^ and ^e^′^,f^′ *p* < 0.001.

**Table 2:** Effects of *Sus scrofa* genotype and sex on craniometric features.

Variables	*Sus scrofa* Groups				*p*-Value			
	LIW	1/4 WB	1/2 WB	3/4 WB	G	S	SW	G x S
GL	22.42 ^e^ ± 0.28	25.31 ^f,e^′ ± 0.23	27.35 ^f,f^′^,e^″ ± 0.26	30.79 ^f,f^′^,f^″ ± 0.58	<0.001	0.001	<0.001	0.489
CBL	24.21 ^e^ ± 0.23	26.35 ^f,e^′ ± 0.19	27.81 ^f,f^′^,e^″ ± 0.22	30.30 ^f,f^′^,f^″ ± 0.47	<0.001	0.002	<0.001	0.288
BL	23.01 ^e^ ± 0.20	24.56 ^f,e^′ ± 0.17	25.85 ^f,f^′^,e^″ ± 0.19	28.70 ^f,f^′^,f^″ ± 0.42	<0.001	<0.001	<0.001	0.277
ZW	13.81 ± 0.11	13.74 ± 0.09	14.01 ± 0.10	14.06 ± 0.22	0.166	0.001	0.003	0.110
OB	9.17 ± 0.35	8.15 ± 0.29	8.06 ± 0.33	8.35 ± 0.73	0.061	0.055	0.743	0.110
PL	11.78 ± 0.14	12.64 ± 0.12	13.39 ± 0.13	15.23 ± 0.29	<0.001	0.004	<0.001	0.524
UTR	6.97 ^a^ ± 0.13	6.68 ^e^ ± 0.11	7.41 ^b,f,e^′ ± 0.12	8.53 ^f,f^′ ± 0.27	<0.001	0.018	0.022	<0.001
MR	4.51 ^c,e^ ± 0.12	4.47 ^c,e^ ± 0.10	4.99 ^d^ ± 0.11	5.63 ^f^ ± 0.25	<0.001	0.982	0.808	0.198
PO	4.16 ± 0.12	4.51 ± 0.10	4.42 ± 0.12	4.32 ± 0.26	0.286	0.828	0.702	0.525
POP	9.74 ± 0.09	9.93 ± 0.08	10.05 ± 0.09	10.15 ± 0.19	0.444	0.001	0.004	0.056
FP	3.82 ± 0.10	4.48 ± 0.08	4.48 ± 0.10	5.34 ± 0.21	<0.001	0.001	0.050	0.157
NL	10.82 ± 0.18	12.36 ± 0.14	13.43 ± 0.17	16.01 ± 0.37	<0.001	0.005	0.001	0.877
OHB	6.65 ^e^ ± 0.10	7.31 ^f,e^′ ± 0.08	7.96 ^f,f^′ ± 0.09	8.19 ^f,f^′ ± 0.20	<0.001	0.020	<0.001	0.018
SH	19.48 ± 0.26	18.99 ^c^ ± 0.22	19.47 ± 0.25	20.63 ^d^ ± 0.55	0.108	0.046	0.580	0.308
LTR	7.27 ^c,e^ ± 0.14	7.75 ^d,e^ ± 0.12	8.38 ^f,c,e^′ ± 0.13	10.04 ^f,f^′ ± 0.29	<0.001	0.939	0.269	0.002
IR	2.06 ^c^ ± 0.08	1.94 ^c^′^,e^ ± 0.07	2.27 ^d^′ ± 0.08	2.63 ^d,f^ ± 0.17	0.002	0.041	0.302	0.659

GL: greatest skull length; CBL: condybasal length; BL: basal length; ZW: bizygomatic width; OB: occipital breadth; PL: palate length; UTR: length of upper tooth row; MR: length of upper molar row; PO: post-orbital breadth; POP: width across post-orbital processes; FP: length of frontal + parietal; NL: nasal length; OHB: occipital height from basion; SH: skull height; LTR: length of lower tooth row; IR: length of upper incisor row; LIW: Lithuanian Indigenous Wattle pigs; 1/4 WB, 1/2 WB and 3/4 WB-LIW: hybrids with 1/4, 1/2 and 3/4 proportions of wild boar; G: genotype; S: sex; SW: skull weight; G x S: genotype x sex interaction. *p*-values of GLM LSD tests for genotype mean values within a row with different superscripts differ significantly at ^a,b^ *p* < 0.05; ^c,d^ *p* < 0.01; ^e,f,^ ^e^′^,f^′ and ^e^″^,f^″ *p* < 0.001.

**Table 3:** Cross validation percentages of discriminant functions between Lithuanian Indigenous Wattle pigs and their hybrids with wild boar.

Segment of Crania		CVP (%)	Predicted Classification			
			LIW	1/4 WB	1/2 WB	3/4 WB
All measurements of whole crania	LIW	90.0	86.7	13.3	0	0
	1/4 WB		7.7	92.3	0	0
	1/2 WB		0	14.3	85.7	0
	3/4 WB		0	12.5	0	87.5
Measurements of length	LIW	90.1	87.5	12.5	0	0
	1/4 WB		7.7	88.5	3.8	0
	1/2 WB		0	9.5	90.5	0
	3/4 WB		0	12.5	0	87.5
Nasal region measurements	LIW	85.9	87.5	12.5	0	0
	1/4 WB		7.7	80.8	11.5	0
	1/2 WB		0	19.0	81.0	0
	3/4 WB		0	0	12.5	87.5
Tooth rows	LIW	64.3	52.3	33.3	13.3	0
	1/4 WB		11.5	69.2	19.2	0
	1/2 WB		14.3	19.0	52.4	14.3
	3/4 WB		0	25.0	0	75.0
Measurements of height	LIW	70.4	56.3	43.8	0	0
	1/4 WB		11.5	80.8	7.7	0
	1/2 WB		0	19.0	76.2	4.8
	3/4 WB		12.5	0	37.5	50.0

CVP: the total correctly assigned cross validation percentages based on measurements used in the discriminant function; LIW: Lithuanian Indigenous Wattle pigs; 1/4 WB, 1/2 WB and 3/4 WB-LIW: hybrids with 1/4, 1/2 and 3/4 proportions of wild boar.

## Data Availability

Data is contained within the article.

## Supplementary Materials

The following are available online at https://www.mdpi.com/article/10.3390/ani13091453/s1, Table S1: The weight of animals and skull measurements.
