# Supplementary material for: Cranial Morphology of Lithuanian Indigenous Wattle Pigs and Their Hybrids with Wild Boar
Source: Animals (Basel). 2023 Apr 24;13(9):1453. doi: 10.3390/ani13091453 (PMC10177289; doi:10.3390/ani13091453)
Supplement: Supplementary file 1 [file animals-13-01453-s001.zip › animals-2315082-supplementary.pdf]

Table S1. The weight of animals and skull measurements

Nr.	Genotype	Lytis/Sex	Svoris/ wheight	Galvos sv/ head	Skull sv	Ap žand sv	GL	CBL	BL	ZW	OB	PL	UTR	MR	PO	POP	FP	NL	OHB	SH	LTR	IR
2830	LV	kiaulaitė	92,0	5,10	0,733	0,234	22,80	23,73	23,77	13,70	13,70	12,30	7,14	4,20	3,40	9,50	4,50	11,15	6,13	18,60	7,10	3,32
3444	LV	kiaulaitė	88,0	5,06	0,917	0,302	22,30	23,90	22,75	14,15	18,00	11,44	7,43	4,49	4,73	10,18	3,42	10,61	6,60	18,10	7,80	2,50
4107	LV	kiaulaitė	88,0	4,86	0,829	0,291	23,54	24,55	23,39	13,59	7,80	11,90	7,10	4,30	3,81	9,72	4,70	11,10	6,80	21,00	7,72	1,17
4998	LV	kiaulaitė	84,0	4,20	0,697	0,229	23,05	24,34	23,00	13,19	7,40	11,84	6,70	4,30	3,60	9,58	4,20	11,00	6,70	19,40	7,00	2,00
6329	LV	kiaulaitė	94,0	5,06	0,790	0,255	24,00	25,39	23,95	13,90	7,60	12,52	7,20	4,31	3,88	9,63	4,12	12,13	7,50	18,90	3,21	x
6332	LV	kiaulaitė	89,0	4,40	0,895	0,293	22,12	24,00	22,55	13,70	7,63	11,50	6,43	4,35	4,80	9,48	3,99	11,10	6,32	20,50	6,70	1,79
6334	LV	kiaulaitė	96,0	5,22	0,871	0,284	22,00	24,62	23,25	13,64	8,03	11,54	6,78	4,45	3,85	9,55	3,17	11,24	6,80	20,90	7,50	1,60
3446	LV	kastratas	90,0	4,74	0,890	0,300	20,61	22,31	21,50	13,22	7,50	11,00	8,00	4,31	4,60	9,04	3,05	10,20	6,30	18,35	7,50	2,65
3441	LV	kastratas	88,0	4,62	0,677	0,216	22,30	23,90	22,62	13,63	8,50	11,70	7,00	4,60	4,80	9,71	3,70	10,23	6,24	18,50	7,79	2,00
3447	LV	kastratas	92,0	4,98	0,864	0,293	21,61	22,90	21,41	14,14	7,90	11,39	6,33	4,10	4,60	9,92	4,30	9,72	7,32	20,80	7,00	1,90
4108	LV	kastratas	95,0	4,88	0,847	0,266	21,21	24,12	22,60	14,15	7,73	11,60	7,30	4,56	4,46	10,20	2,91	10,40	6,80	19,00	7,42	1,95
4109	LV	kastratas	92,0	5,24	0,871	0,283	21,82	24,24	22,80	13,90	7,85	11,12	7,20	4,50	4,52	9,90	3,45	10,20	6,75	18,00	7,50	1,95
6330	LV	kastratas	92,0	5,46	0,779	0,266	23,20	25,40	23,82	14,41	7,85	12,50	7,14	4,50	3,69	9,75	3,60	11,10	6,48	19,70	6,65	2,10
6331	LV	kastratas	93,4	5,26	0,915	0,290	24,30	25,84	24,30	13,67	7,75	12,80	6,90	4,55	4,00	9,91	4,23	11,74	6,86	18,54	7,24	2,24
6336	LV	kastratas	95,0	5,66	1 003	0,314	22,73	25,41	24,16	14,26	8,10	12,32	6,65	6,39	4,25	10,30	3,85	11,10	7,10	21,14	6,39	1,90
6339	LV	kastratas	95,0	5,30	0,861	0,285	22,20	24,20	23,09	14,20	8,00	11,84	6,60	4,60	3,62	9,60	3,65	10,80	6,85	18,96	7,60	1,79
be nr	LV	kastratas			0,573	0,183	21,60	22,19	21,75	12,89	7,30	10,99	6,22	5,40	3,50	8,88	3,65	10,52	6,50	18,34	6,96	1,50
2174	H25%	kiaulaitė	87,4	5,65	0,997	0,309	23,78	24,50	23,19	14,39	8,00	11,89	6,40	4,50	4,90	10,12	4,09	11,30	6,92	18,67	7,65	1,60
3438	H25%	kiaulaitė	88,6	5,10	0,954	0,305	25,10	25,73	24,24	13,75	8,75	12,43	6,40	4,36	4,32	10,24	4,85	12,24	7,60	19,55	7,67	1,79
3440	H25%	kiaulaitė	85,0	4,72	0,777	0,264	25,40	26,14	24,74	13,25	8,18	12,80	6,30	4,29	4,29	9,39	4,10	13,21	7,29	18,00	6,65	1,79
3442	H25%	kiaulaitė	86,6	4,66	0,877	0,289	25,10	25,81	24,50	13,50	8,24	12,24	6,70	4,70	4,80	9,60	4,89	11,74	7,00	19,24	7,50	1,86
4111	H25%	kiaulaitė	85,1	4,90	0,787	0,255	23,90	25,60	23,56	13,20	8,10	11,85	6,20	4,24	3,99	10,00	4,43	11,67	7,49	19,54	7,70	1,60
5001	H25%	kiaulaitė	95,2	5,24	1 007	0,335	25,80	26,60	24,48	14,15	8,10	12,84	6,49	4,49	4,48	10,17	4,95	12,50	7,60	19,90	7,79	1,71
5004	H25%	kiaulaitė	89,2	4,86	0,979	0,289	26,50	26,40	24,86	13,40	8,00	12,67	6,24	4,20	5,24	9,99	5,40	13,20	7,80	18,60	7,55	1,55
5005	H25%	kiaulaitė	86,4	4,76	0,760	0,232	24,55	25,00	23,65	13,62	7,70	11,79	6,50	4,50	4,32	9,43	4,00	11,70	7,20	17,80	7,70	1,90
4996	H25%	kiaulaitė	86,0	4,86	0,926	0,297	25,30	26,10	24,40	13,10	7,80	12,24	6,50	4,50	4,70	9,65	4,40	12,67	7,40	19,35	7,80	1,70
2171	H25%	kastratas	91,0	5,95	0,958	0,288	23,69	25,10	23,40	14,20	8,58	11,80	6,70	4,88	4,76	10,14	4,40	11,60	7,09	20,24	8,10	1,72
2172	H25%	kastratas	87,0	5,20	0,808	0,257	24,67	26,24	24,29	12,64	7,30	12,50	7,08	4,50	3,90	9,08	4,30	12,48	6,60	20,25	7,89	1,80
2173	H25%	kastratas	92,4	5,65	0,942	0,296	24,08	26,10	24,14	14,06	8,16	12,48	6,44	4,59	4,99	10,24	3,58	12,06	7,70	20,34	7,75	2,09
2828	H25%	kastratas	88,0	5,25	0,792	0,271	25,24	26,40	24,55	13,13	7,70	12,50	7,05	4,40	4,00	9,19	4,20	12,30	6,85	18,40	7,50	1,80

3449	H25%	kastratas	83,0	4,44	0,822	0,255	24,20	25,24	23,58	13,27	7,20	12,20	6,90	4,65	4,89	9,20	4,20	11,40	7,00	16,90	7,68	1,75
4110	H25%	kastratas	86,3	5,08	0,949	0,311	24,20	25,75	24,00	13,10	8,08	11,70	6,52	4,40	5,12	9,74	4,10	11,74	7,09	18,54	8,20	1,42
4112	H25%	kastratas	88,1	4,40	0,735	0,230	24,24	25,36	23,40	13,50	7,84	12,42	6,80	4,70	4,75	10,00	4,40	11,75	6,99	18,67	7,90	1,80
4116	H25%	kastratas	90,0	4,48	0,870	0,289	22,62	24,35	22,89	13,04	8,20	11,50	6,35	4,43	4,49	9,43	3,80	10,65	6,24	18,14	7,50	1,70
5006	H25%	kastratas	97,4	4,62	0,751	0,242	24,80	25,40	23,24	12,79	7,50	12,10	6,30	4,40	4,20	9,30	4,39	11,79	7,60	19,30	7,70	1,89
4995	H25%	kastratas	87,2	4,66	0,855	0,254	25,84	26,50	24,70	12,75	7,85	12,81	6,55	4,50	4,60	9,65	4,40	12,62	7,50	18,70	8,40	2,10
4997	H25%	kastratas	86,0	4,72	0,893	0,276	24,92	25,71	23,80	13,91	7,95	12,70	6,50	4,50	4,40	9,43	4,10	12,30	7,05	18,62	7,09	2,00
6337	H25%	kastratas	89,5	4,44	0,860	0,260	24,64	26,35	24,41	13,67	7,80	13,00	6,64	4,74	4,00	9,52	4,49	12,16	7,54	19,00	8,14	2,24
1541	H25%	kuiliukas	88,4	5,80	0,953	0,305	25,31	26,16	24,45	14,50	8,49	12,58	6,60	4,55	4,72	10,12	4,16	12,20	7,35	19,13	7,33	2,18
1542	H25%	kuiliukas	91,2	7,10	1 188	0,382	24,80	25,55	24,34	14,90	8,33	12,20	6,86	4,80	5,45	10,80	4,86	11,95	6,79	20,25	8,00	2,31
2166	H25%	kuiliukas	91,0	5,98	0,898	0,283	26,70	27,95	25,74	13,58	8,36	13,39	7,19	4,49	4,20	9,81	4,40	13,66	7,12	18,39	7,54	1,86
2827	H25%	kuiliukas	88,4	5,15	0,820	0,260	25,55	27,14	24,79	13,35	8,16	12,85	6,75	4,25	3,92	9,92	4,50	12,53	7,18	18,28	7,84	2,24
2829	H25%	kuiliukas	88,8	5,75	0,889	0,293	25,98	26,87	25,30	14,00	8,75	13,35	6,45	4,18	4,22	10,24	4,75	12,20	7,35	18,89	7,60	2,25
2167	H50%	kuiliukas	93,0	5,52	0,991	0,335	27,95	28,08	25,74	14,20	8,60	13,39	6,99	4,79	3,43	9,99	5,05	13,40	8,24	20,08	7,50	1,90
9967	H50%	kuiliukas	93,2	6,36	1 011	0,314	27,62	27,90	26,57	13,85	7,67	13,70	6,72	4,73	4,69	9,54	4,61	13,62	8,05	20,10	8,71	2,60
9968	H50%	kuiliukas	84,2	6,54	0,874	0,271	26,20	26,24	25,02	13,89	7,75	13,15	6,89	4,60	4,35	9,45	4,29	13,70	7,59	18,70	7,60	2,21
9970	H50%	kuiliukas	85	4,98	0,975	0,294	26,90	27,10	25,10	14,26	7,65	13,20	7,10	6,67	5,00	9,85	4,50	13,20	7,80	19,14	6,85	2,29
9972	H50%	kuiliukas	89	5,56	1 015	0,309	28,62	27,84	26,30	14,30	8,20	13,58	6,99	4,80	5,15	10,50	5,10	13,50	8,17	18,45	7,90	2,15
1097	H50%	kuiliukas	88,4	5,6	0,977	0,305	27,62	27,76	26,34	13,97	7,91	13,79	6,68	4,60	4,36	9,87	4,30	14,00	8,15	18,93	8,47	2,57
1098	H50%	kuiliukas	90,2	6,26	1 016	0,326	27,00	28,05	26,40	14,20	8,12	13,58	7,74	4,62	4,30	10,30	4,50	13,52	8,00	21,52	7,65	2,60
1099	H50%	kuiliukas	88,6	6,3	0,994	0,316	27,72	28,84	26,43	14,10	8,33	13,98	6,77	4,69	4,80	10,59	4,70	13,25	8,29	19,30	7,90	2,59
2168	H50%	kuiliukas	93	6,3	1 157	0,353	27,79	27,31	26,00	14,58	8,12	13,59	6,70	4,59	4,40	10,42	4,60	13,90	8,82	19,72	8,04	2,20
H1	H50%	kuiliukas			0,855	0,286	28,40	29,20	26,74	13,48	7,99	13,11	6,86	4,74	4,23	9,70	4,80	14,12	8,05	20,45	6,62	2,24
9969	H50%	kastratas	92	5,86	0,882	0,272	26,15	27,00	24,83	13,80	7,60	12,81	6,80	4,85	4,35	9,70	4,50	12,66	7,35	17,30	7,40	1,99
9971	H50%	kastratas	98	5,32	0,824	0,269	27,10	27,99	25,80	13,55	7,85	13,60	6,74	4,70	4,10	9,58	4,49	13,39	7,65	18,55	8,10	2,30
1096	H50%	kastratas	85,6	5,48	0,942	0,288	25,83	26,65	25,05	13,39	8,10	12,91	6,70	4,80	3,91	9,45	3,79	12,70	7,38	19,10	7,50	2,14
1100	H50%	kastratas	90,8	5,92	0,875	0,281	26,70	27,43	25,31	14,10	7,99	13,28	6,60	4,83	4,22	9,43	4,20	12,89	8,24	19,67	7,65	2,60
1543	H50%	kastratas	92,4	5,55	0,994	0,311	27,70	27,80	26,01	13,41	8,28	13,79	6,53	4,63	4,39	9,86	4,33	13,70	7,86	18,80	8,65	2,35
2170	H50%	kastratas	93	5,95	0,868	0,267	27,12	27,40	24,90	13,48	8,04	12,79	6,90	4,70	4,00	9,59	4,71	12,81	7,90	18,10	8,00	1,80
2169	H50%	kastratas	93	5,72	1 014	0,303	26,60	26,62	25,19	13,60	7,98	13,06	6,60	4,80	4,80	9,89	4,41	13,05	8,20	18,20	8,00	2,60
3437	H50%	kastratas	89	5,34	1 076	0,370	28,11	28,15	26,53	14,40	8,13	13,55	6,80	4,72	3,77	10,05	4,60	13,67	8,40	19,60	8,05	2,20
4994	H50%	kiaulaitė	97	5,12	1 099	0,371	27,33	28,90	26,62	14,20	8,46	14,00	9,24	5,65	4,20	10,48	4,40	14,24	7,90	20,40	9,39	2,20
4993	H50%	kiaulaitė	95	5,64	1 311	0,415	28,52	28,90	26,50	14,70	8,24	13,80	8,50	5,30	5,19	10,80	4,17	13,73	8,20	19,80	9,97	2,20
4999	H50%	kiaulaitė	94	5,28	1 116	0,335	28,10	28,20	26,10	14,15	7,84	13,22	8,36	5,00	4,35	10,30	4,91	13,40	8,09	20,30	9,50	2,40

7079	H75%	kiaulaitė	111	9,26	1 240	0,405	31,93	31,40	29,55	13,90	7,98	15,70	9,60	5,79	4,00	9,99	5,37	16,10	8,45	21,30	10,60	2,50
7081	H75%	kiaulaitė	101	8,52	1 311	0,403	33,30	32,00	30,22	14,20	8,29	16,00	9,70	6,00	4,22	10,55	5,64	17,60	8,48	19,60	10,62	2,70
12322	H75%	kiaulaitė	112	8,00	1 412	0,466	33,43	33,43	31,30	14,60	8,29	17,05	9,21	5,34	3,48	10,30	5,00	18,20	8,50	20,70	10,00	2,90
2422	H75%	kiaulaitė	104	7,47	1 251	0,358	29,80	29,60	27,43	13,92	9,53	14,39	6,54	4,50	5,30	11,00	4,92	15,55	8,55	22,55	8,30	2,12
3849	H75%	kiaulaitė	87,3	6,22	0,825	0,266	25,24	26,58	25,39	13,48	8,43	12,81	6,75	4,34	4,00	9,23	4,50	12,95	6,09	19,85	7,60	2,00
4877	H75%	kiaulaitė	85,6	6,14	0,953	0,299	29,10	28,76	27,00	13,22	8,30	14,79	8,41	6,54	4,33	10,00	4,40	15,81	7,85	22,74	9,45	2,50
4878	H75%	kiaulaitė	84,3	6,1	0,886	0,289	29,65	28,15	26,70	13,34	7,95	14,10	7,49	5,48	3,70	9,70	4,95	15,70	7,46	23,04	9,90	2,30
7080	H75%	kuiliukas	93	8,8	1 288	0,429	32,30	31,74	30,12	14,60	8,40	16,05	9,08	5,80	4,45	10,45	5,90	16,59	8,90	20,00	10,89	2,90
